# Trace membrane additives affect lipid phases with distinct mechanisms: a modified Ising model

**DOI:** 10.1007/s00249-015-1017-x

**Published:** 2015-03-28

**Authors:** Rebecca L. Meerschaert, Christopher V. Kelly

**Affiliations:** Department of Physics and Astronomy, Wayne State University, Detroit, MI USA

**Keywords:** Lipid membrane, Phase transition temperature, Miscibility, Correlation length, Phase partitioning, Simulation

## Abstract

The addition of trace molecules into membranes can significantly alter the morphology of the co-existing liquid phases and lipid phase transition temperature. Membrane additives may affect lipid phase dynamics through preferentially partitioning to the boundary between lipid phases or preferentially mixing into one lipid phase. The characteristic differences between these mechanisms are demonstrated here in a minimalistic nearest neighbor model to provide a framework for how slight changes to membrane composition may affect lipid-phase-dependent processes, such as lipid-raft formation, immunological signaling, and molecular sorting preceding endocytosis with coexisting liquid phases. Within the low mole fractions explored here (≤3 mol%), increasing the additive concentration linearly changed the phase miscibility temperature. Rotationally asymmetric Janus particles reduced the miscibility transition temperature for all fractions and degree of phase polarization. Rotationally symmetric additives, however, either increased or decreased the phase miscibility temperature depending on the phase preference of the additive. While most experimental molecules may contain aspects of both of these idealized additives, this model provides a broad framework to quantify the effects of membrane additives in regard to lipid phase preference, lipid-raft association, and contribution to lipid phase-dependent molecular sorting.

**Electronic supplementary material:**

The online version of this article (doi:10.1007/s00249-015-1017-x) contains supplementary material, which is available to authorized users.

## Introduction

The presence of coexisting liquid-ordered and liquid-disordered lipid phases has been implicated in diverse biological processes with high sensitivity to perturbations in membrane composition, tension, curvature, and temperature (Lingwood et al. 2008). Lipid phase dynamics may inherently provide nanoscale clustering and facilitate lipid-raft-mediated processes (Veatch et al. 2008). However, trace membrane additives can disturb the lipid phase dynamics and the associated biological processes. Some additives possess rotational asymmetry, preferentially localize to the phase boundary, reduce the phase miscibility, decrease the miscibility transition temperature (*T*_mis_), and act as linactants, 1D analogs of surfactants (Trabelsi et al. 2008). Other membrane additives are rotationally symmetric, preferentially localize into one particular lipid phase, and may increase or decrease *T*_mis_. Broadly, the effects of membrane additives are quantified by the change to the lipid phase *T*_mis_. The difference between a sample temperature and *T*_mis_ determines fundamental equilibrium qualities such as the correlation length of the coexisting phases (Honerkamp-Smith et al. 2008; Palmieri and Safran 2013a) and the dynamics of the phase mobility (Honerkamp-Smith et al. 2012; Palmieri and Safran 2013b).

Numerous molecules have demonstrated potent abilities to alter membrane phase miscibility without necessarily containing a dependence on the rotation of the molecule within the bilayer, including cholesterol (Dietrich et al. 2001; Zhao et al. 2007a; Levental et al. 2009; Heberle et al. 2010), fluorophores (Veatch et al. 2007a), anesthetics (Jorgensen et al. 1991; Gray et al. 2013), insecticides (Jorgensen et al. 1991), *n*-propyl gallate (Zhao et al. 2007b), C-reactive protein (Sáenz et al. 2010), and flavonoids (Ostroumova et al. 2014). Other molecules have been hypothesized to prefer the boundary between two lipid phases because of their molecular structure; for example, hybrid lipids contain one liquid-order-preferring tail and one liquid-disorder-preferring tail (Brewster et al. 2009; Hassan-Zadeh et al. 2014; Li and Gorfe 2014), and asymmetrically lipidated proteins, such as N-Ras and H-Ras, have shown preference for partitioning at the lipid phase boundary (Nicolini et al. 2005; Janosi et al. 2012; Li et al. 2012). Further, molecules such as the nonsteroidal anti-inflammatory drug indomethacin may contain both a net phase preference and significant molecular structure asymmetry, although the nanoscale partitioning has yet to be studied (Zhou et al. 2010).

Previous studies have focused on theoretical predictions and experimental examination of systems with a significant fraction of the membrane consisting of phase-polarized particles (>10 mol%) (Brewster et al. 2009; Yamamoto et al. 2010; Yamamoto and Safran 2011; Palmieri and Safran 2013a, b; Hassan-Zadeh et al. 2014; Palmieri et al. 2014), whereas this manuscript focuses on small composition changes that can greatly affect membrane phase dynamics and provide a means for live cells to efficiently regulate lipid phase-dependent processes.

The morphology of lipid mixture phase transitions is of the Ising universality class. Monte Carlo simulations of 2D Ising models with conserved order parameters have been used to demonstrate key membrane properties (Frazier et al. 2007; Yethiraj and Weisshaar 2007; Honerkamp-Smith et al. 2008; Machta et al. 2011, 2012). Within the Ising model, a particle’s phase preference (*σ*) is quantified for liquid-disordered (black*, σ* = −1) or liquid-ordered (white*, σ* = 1) phases, although the particular phase associated with the color and sign of *σ* is arbitrary in this symmetric system. The internal energy between two particles (*J*_*i,j*_) is equal to *J* for nearest neighbors and zero otherwise, and it can be calculated from the sum of all particle interactions in the Hamiltonian (*H*) according to:

1 $$H = -\sum\limits_{ \langle i, j \rangle } {J_{i,j } \sigma_{i} \sigma_{j} } .$$

When this model contains 50 % white and 50 % black particles on a square lattice, it exhibits critical behavior with $T_{\text{mis}} = 2J/(k_{\text{B}} \ln (1 + \sqrt 2 ))$, where *k*_B_ is Boltzmann’s constant. Isolated plasma membrane vesicles from mast cells happen to be of a composition that demonstrates near-critical behavior with *T*_mis_ = 22 °C (Veatch et al. 2008), corresponding to a value of *J* = 0.26 kcal/mol. To relate the complex molecular composition of near-critical membranes to the two-state Ising model, each pixel from the model represents the mean composition of each state. For example, the white particles in the Ising model represent the cell membrane’s average liquid-ordered phase composed of lipids with longer, more saturated acyl tails, greater sphingomyelin content, and slightly higher cholesterol content than the black particles. This model permits the observation of complex phase behaviors without explicit incorporation of the molecular details or concentrations of the membrane, as long as the average phase segregation preferences are represented by the value of *J*.

**Fig. 1:**
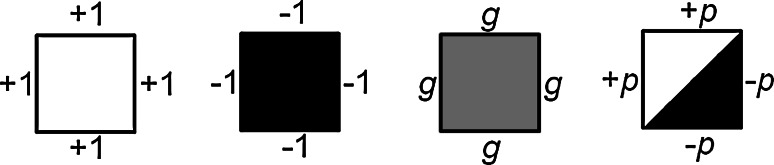
*White*, liquid-ordered (*σ* = 1); *black*, liquid-disordered (*σ* = −1); *gray particles* (*σ* = *g*) were rotationally symmetric. Phase-polarized additives, or Janus additives, were rotationally asymmetric with a polarized phase preference (|*σ*| = *p*)

In this manuscript, we demonstrate the influence of trace additives in a two-component nearest neighbor model on a square lattice with conserved order parameters as a means of simulating how additives of differing molecular structure could influence lipid phase mixing. Instead of modifying the value of *J* to represent changes in membrane composition, a third particle type was incorporated in these simulations to provide greater structural detail of the additive’s effect on phase separation and additive partitioning. In a ternary lipid membrane, for example, the additive could be a fourth type of molecule or more of one molecule type that was already present in the membrane. Simulated additives were either rotationally symmetric gray particles or rotational asymmetric phase-polarized particles with gray values of *g* or polarizations of *p*, respectively (Fig. 1). Generally, the addition of a third type of particle to the system results in a change to the phase diagram, new phase transitions, and variations from critical behavior. Simulations performed with additives of *g* = 0 were analogous to the Blume-Emery-Griffiths model (Blume et al. 1971), for which the phase transitions were modeled against an additive concentration with mean-field approximations. However, the additive concentrations explored here are consistent with the composition fluctuations in living and model membranes, including variations in content of cholesterol (Dietrich et al. 2001; Pokorny et al. 2006; Frazier et al. 2007; Zhao et al. 2007a; Levental et al. 2009; Heberle et al. 2010), fluorophores (Veatch et al. 2007a; Frazier et al. 2007), and a variety of other biologically active molecules (Jorgensen et al. 1991; Nicolini et al. 2005; Pokorny et al. 2006; Zhao et al. 2007a, b; Brewster et al. 2009; Sáenz et al. 2010; Zhou et al. 2010; Gray et al. 2013; Ostroumova et al. 2014). While real membranes have been demonstrated to be near critical in composition (Veatch et al. 2007b, 2008), they have not been shown to be any closer to critical than the additive-included simulations shown here. Further, the experimental method for observing plasma membrane extracts involved the incorporation of fluorescent lipid additives (<0.25 mol%) (Veatch et al. 2008), which altered the membrane composition and presumably its phase behavior but did not diminish the significance or confidence in the result that the membranes were near critical in composition.

This work focuses on small perturbations to the well-understood two-state Ising model and demonstrates how trace additives would affect the characteristic size of the co-existing single-phase domains (*ξ*) and *T*_mis_. For ≤3 mol% additives, *T*_mis_ and ξ both changed monotonically with increasing additive concentration, while *T*_mis_ changed linearly. Rotationally symmetric particles of *g* < 1 and rotationally asymmetric particles of all values of *p* caused a decrease in both *T*_mis_ and *ξ*. However, rotationally symmetric additives of *g* > 1 resulted in an increase in both *T*_mis_ and *ξ*. The morphological detail provided by this model yields the distributions of additives within the system, which is unavailable from mean-field models. The resulting distributions of the additives at the boundary between phases were quantified, and the applicability of this analysis for assessing experimental membrane additives or cellular perturbations is discussed.

## Methods

Non-local particle exchanges were allowed via a Monte Carlo algorithm conserving the system composition (e.g., 49.5 % white, 49.5 % black, and 1 % additive) and equilibrated for 10^5^ global equilibration sweeps on a 512 × 512 square grid with biperiodic boundary conditions. An additional 10^5^ global sweeps were performed over which an average of the system parameters was reported, e.g., the correlation length. The symmetry of this configuration with equal fractions of white and black particles resulted in the additives having the same effect, regardless of the sign of *g* or *p.* Further details are provided in the Supplemental Material including a movie of 50 different time points of the equilibrated system.

**Fig. 2:**
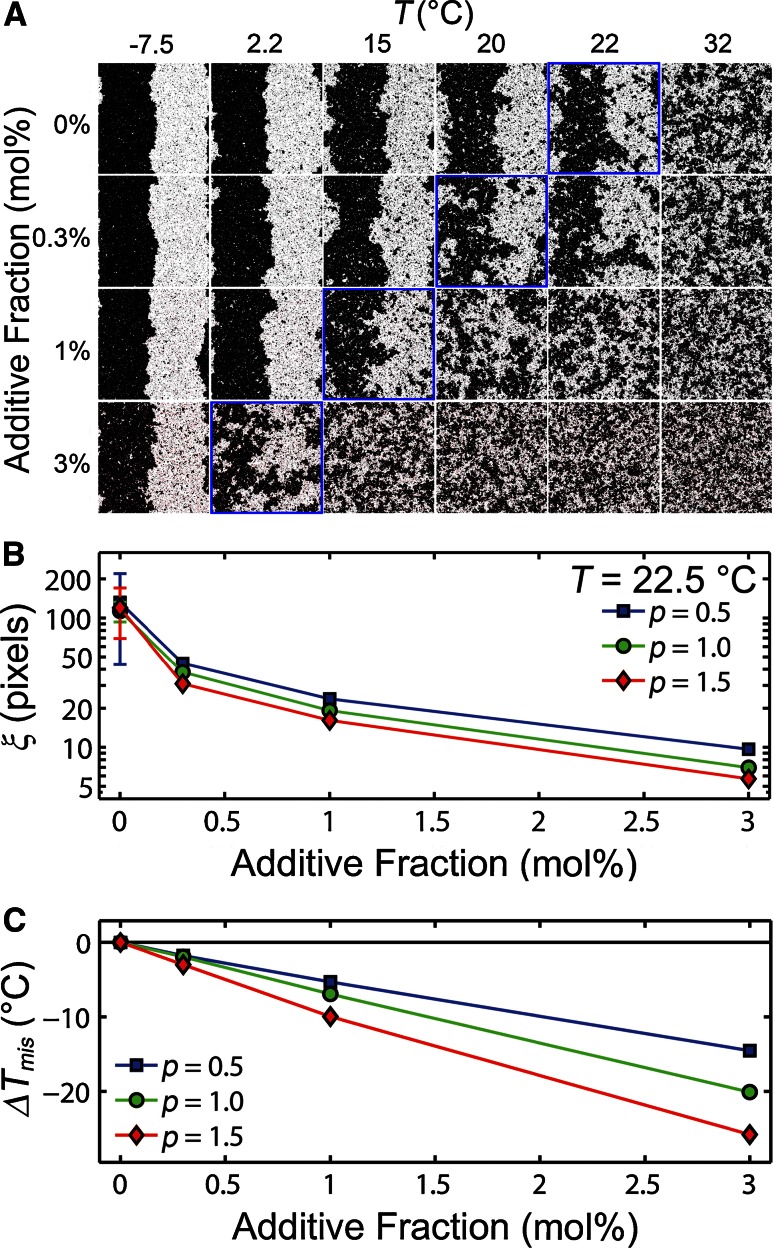
Increasing the mole fraction of phase-polarized particles caused an increase in the phase miscibility and a decrease of *T*_mis_. **a** With *p* = 1, phase-polarized additives are displayed as *red squares* in these images, and *zoomed-in regions* show the individual additives in Fig. S5. *Blue outlined images* indicate where *T* = *T*_mis_ with *J* set such that *T*_mis_ of the additive-free system $(T_{mis}^{\prime})$ is 22 °C. Increasing the concentration of phase-polarized particles with *p* = 1 resulted in a decrease of **b** *ξ* and **c** *T*_mis_. Δ*T*_mis_ = *T*_mis_−$T_{mis}^{\prime}$. Uncertainty of $T_{mis}^{\prime}$ was approximately 0.6 °C, and uncertainty of ξ was smaller than the symbol unless otherwise indicated

The correlation length of the phases was calculated from the azimuthal average of the 2D spatial correlation of the system configuration (*I*) where all white particles were valued at 1, black particles at 0, and additives at (*g*/2 + 0.5). Unless otherwise stated, if *p* ≠ 0, then *g* = 0, and vice versa. The 2D correlation function (*C*) as a function of the position $\left( {\bar{r} } \right)$ can be calculated by

2 $$C\left( {\bar {r} } \right) = \frac{{\left\langle {I\left( {\overline{R} } \right)I\left( {\overline{R} + \overline{r} } \right)} \right\rangle }}{{\left\langle {I\left( {\overline{R} } \right)} \right\rangle^{2} }}, \,$$

where $\langle \, \rangle$ represents the average over all 2D space $( {\bar {R} })$. Computationally, this was expedited by the use of fast Fourier transforms (FFTs) with

3 $$C\left( {\bar {r} } \right) = \frac{{{\text{FFT}}^{ - 1} \left( {\left| {{\text{FFT}}\left( I \right)} \right|^{2} } \right)}}{N},$$

and normalization (*N*) (Veatch et al. 2012). The 1D correlation function (*c*) was computed from

4 $$c\left( r \right) = \frac{1}{2\pi }\int\limits_{0}^{2\pi } {C\left( {r,\theta } \right){\text{d}}\theta } ,$$

and the correlation length (*ξ*) was calculated by nonlinear least square fitting of *c*_fit_ to *c*(*r*) where

5 $$c_{\text{fit}} = c_{0} \frac{{{\text{e}}^{ - r/\xi } }}{{r^{\eta } }}$$

and the critical exponent *η* equals 0.25, which is exact for the Ising model. The critical exponents for the three-component systems and finite systems are not equal to that of the Ising model; however, additionally allowing *η* to change did not significantly change fitting results.

Changes to *T*_mis_ were measured for various additive fractions and additive types in this minimalistic model. Δ*T*_mis_ was determined by measuring the shift in *ξ* as a function of temperature and by ascertaining the maximum of the specific heat versus temperature (Veatch et al. 2012) (Figs. S1–S3). The finite size effects that alter the perceived transition temperature were consistent for all of these simulations on the same size system, which permitted this simplistic calculation of Δ*T*_mis_ and its broad applicability to systems of other sizes. A demonstration of the independence of these results on system size is presented in the Supplemental Material (Fig. S4).

## Results

**Fig. 3:**
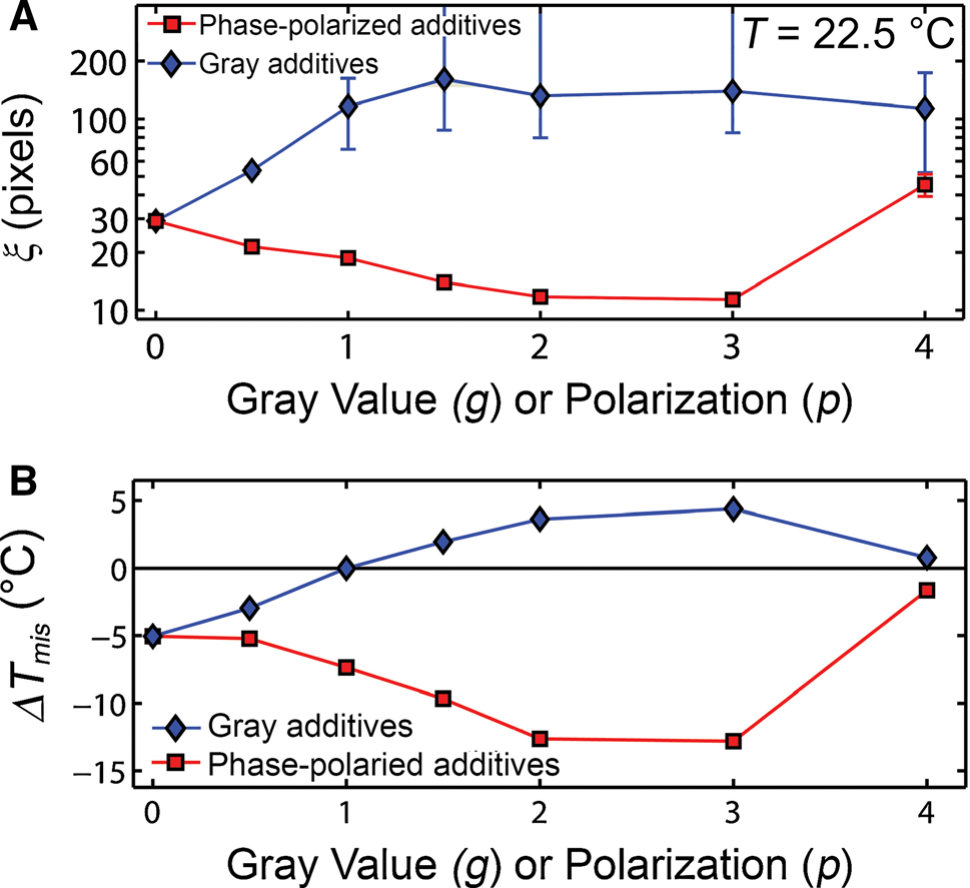
**a** *ξ* and **b** *T*_mis_ and either increased or decreased upon additive addition depending on the additive properties. Δ*T*_mis_ = *T*_mis_−$T_{mis}^{\prime}$ where $T_{mis}^{\prime}$ = 22 °C. Additives comprised 1 mol% of the total particles in these simulations. Measurement of *ξ* is limited by the system size, resulting in large uncertainties for situations with *ξ* > 100 pixels (Fig. S4). Images of these systems are included in the Supplemental Material (Figs. S5, S6). Δ*T*_mis_ and ξ changes are symmetrical for negative values of *g* or *p*

Changes in phase miscibility with an increasing additive fraction are visibly obvious in images of the system configuration (Figs. 2, S5, S6). *T*_mis_ decreased linearly with increasing fractions of phase-polarized particles, or Janus particles, as predicted from mean-field models (Yethiraj and Weisshaar 2007; Brewster et al. 2009). The miscibility temperature is reduced for any phase polarization (*p*), while rotationally symmetric gray particles either increased or decreased *T*_mis_ depending on their gray value (*g*) (Figs. 3, S6). Increasing *p* for the phase-polarized additives resulted in a greater decrease in *T*_mis_ until *p* > 3, upon which the additives segregated into a third phase, separate from the white and black particles, and reduced the white and black particle mixing. Gray particles with *g* < 1 encouraged phase miscibility by decreasing the differences between the ordered and disordered phases, analogous to the incorporation of *n*-alcohols into membranes (Gray et al. 2013). However, when *g* > 1, the opposite effect dominates; the difference between the phases was increased when *g* > 1 and *T*_mis_ was increased, analogous to the addition of δ-lysin to membranes (Pokorny et al. 2006). Gray particles with *g* > 1 were no longer of in-between phase and were more liquid-order preferring than white particles, i.e., “super-white.”

When *p* or *g* > 3, the additives condensed and formed a distinct phase excluding white or black particles (Fig. S6), and the white-black miscibility temperature became less affected by the additives (Fig. 3). For *p* or *g* > 3, simulations were unable to capture a diverse set of configurations for the additive aggregation because of the improbable rearrangement of the condensed phases, analogous to the kinetically trapped configurations that are commonly experienced experimentally. Aggregates of phase-polarized particles at *p* = 4 demonstrated rotational ordering of the additives (Fig. S7), which resulted in slower additive diffusion.

**Fig. 4:**
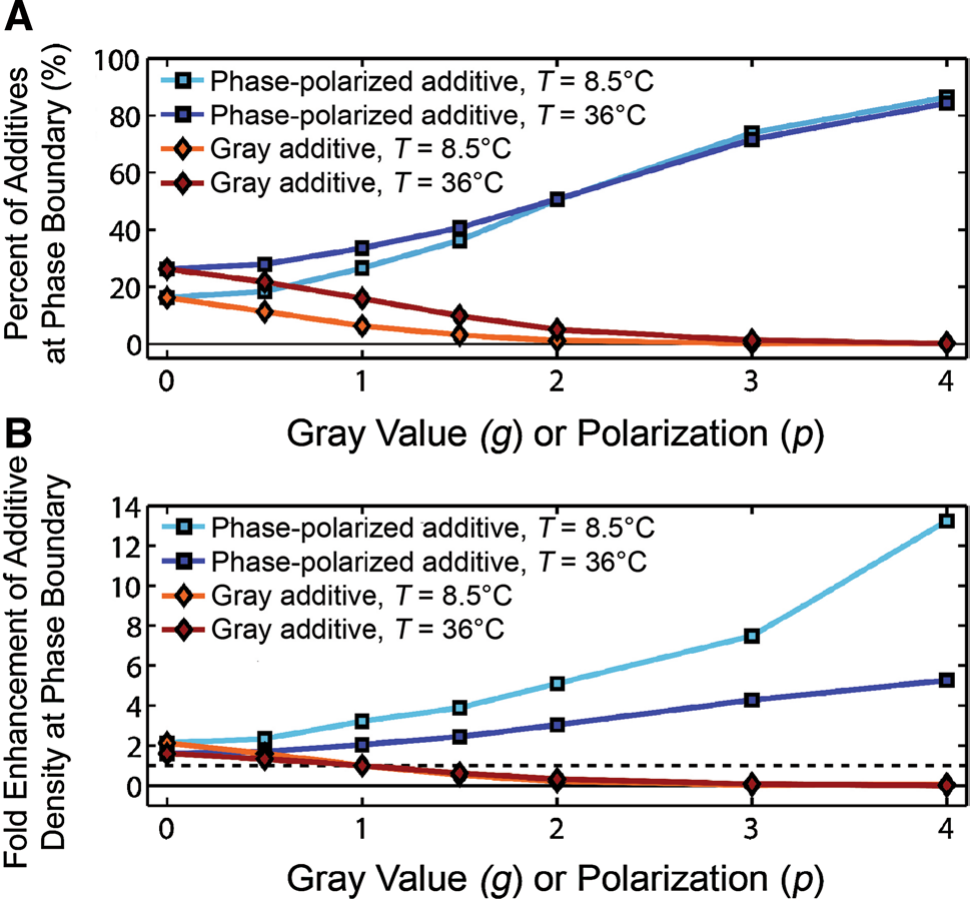
**a** The probability that an additive was found at the phase boundary varied with the additive properties and temperature. The fraction of additives at the boundary was calculated as the ratio of the number of additives surrounded by two white and two black particles versus the total number of additives, excluding additives adjacent to other additives. **b** The additive locations were normalized by the amount of phase boundary in each condition to yield the fold enhancement of the additive at the phase boundary versus generic white particles. Additives were present at 1 mol%. Uncertainty was calculated as the standard deviation of different time points and smaller than the symbol unless otherwise indicated

The partitioning of additives in each phase and at the domain boundary was measured. A particle was declared to be at the phase boundary if it was adjacent to two white and two black particles. The ratio of additives at the phase boundary to all additives with no adjacent additives was calculated (Fig. 4a). As *p* increased, the fraction of phase-polarized particles at the phase boundary increased; as *g* increased, the fraction of gray particles at the phase boundary decreased. Lowering the temperature would have made phase-polarized particles more likely to be found at the phase boundary if the phase morphology had stayed consistent. However, increasing temperature from 8.5 to 36 °C resulted in 2.3 ± 0.4 times more phase boundary (Fig. S8), which was dependent on both simulation size and additive properties, and generally resulted in a higher probability for the additives being located at the phase boundary for higher temperatures. Additive partitioning was normalized to the amount of phase boundary in order to calculate the fold enhancement of the additive to the phase boundary relative to the white or black particles (Fig. 4b). Additives more concentrated at the phase boundary than the *g* = 1 particles preferentially partitioned at the phase boundary, whereas additives less concentrated at the phase boundary than the *g* = 1 particles preferentially partitioned away from the phase boundary, which is consistent with the sign of Δ*T*_mis_ (Fig. 3). Although there is not a local energy difference for the *g* = 0 additive if it is immersed in a single phase or at the phase boundary, there is an energy difference for the system that encourages *g* < 1 additives to be concentrated at the phase boundary. At colder temperatures, the phase-polarized additives especially concentrated at the phase boundary because of the decreased entropic drive for mixing.

These results apply to additives in systems that are near critical compositions. It is important to note that these results would vary greatly for compositions that are far from critical compositions because of the increased phase boundary line tension and the increased likelihood for boundary-preferring particles to localize at the boundary in such systems.

**Fig. 5:**
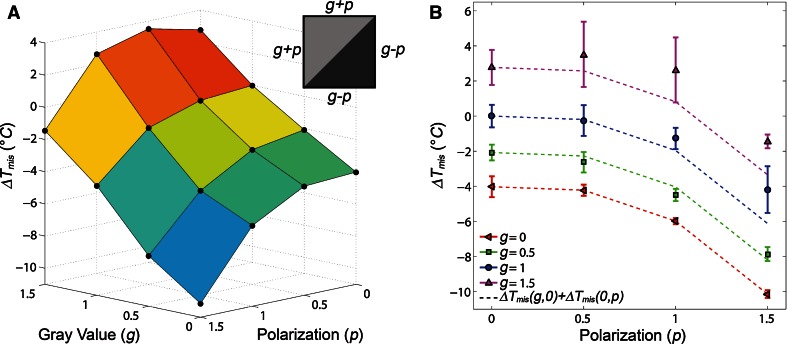
**a** Changes to the miscibility transition temperature were induced by the incorporation of 1 mol% additives with both net and rotationally asymmetric phase preferences, i.e., dependent on both *g* and *p* values, as shown in the inset. **b** For small values of *g* and *p*, Δ*T*_mis_ as a function of *g* and *p*, Δ*T*_mis_ (*g*, *p*), is approximately equal to Δ*T*_mis_ (*g*, 0) + Δ*T*_mis_ (0, *p*)

In all the prior described simulations, only additives with either *g* ≠ 0 or *p* ≠ 0 were considered. However, real membrane additives would likely possess both a net phase preference (*g* ≠ 0) and a rotational phase asymmetry (*p* ≠ 0). To predict how changing both *g* and *p* for a membrane additive would affect the miscibility transition temperature of the membrane, simulations were performed for additives with rotational asymmetry and a net phase preference at 1 mol% (Fig. 5). The changes to the miscibility transition temperature were dependent on both the *g* and *p* value of the additive in a cumulative effect. For small values of *g* and *p* (i.e., either *g* or *p* ≤ 1), Δ*T*_mis_ as a function of *g* and *p* was approximately equal to the sum of the change in miscibility temperature from 1 mol% gray particles of gray value *g* plus the change from 1 mol% polarized particles of polarization *p* (Fig. 5b).

## Discussion

Taken together, these idealized membrane additives represent different means by which molecules may alter the lipid phases in a membrane. A membrane-associated molecule may be experimentally quantified with both a *p* value and *g* value to describe the effects of the additive molecular structure on lipid phase dynamics in a particular membrane. *g* is analogous to the partition constant and quantifies the molecule’s preference for one phase over the other. *p* quantifies the molecule’s phase asymmetry and preference to partition at the phase boundary. The effects of a particular membrane additive are determined entirely by the membrane’s additive-free miscibility temperature ( $T_{mis}^{\prime}$) or internal energy between two particles (*J)* of the near-critical membrane without a dependence on the particular molecular details of the individual membrane components. The temperatures reported throughout this article could be alternately represented as reduced temperatures (*T*_R_), where *T*_R_ = (*T* −  $T_{mis}^{\prime}$)/ $T_{mis}^{\prime}$. Upon rescaling, these results would be directly applicable to other membranes with different values of *J* to predict Δ*T*_mis_ for different $T_{mis}^{\prime}$. Further, experimental use of membranes of varying $T_{mis}^{\prime}$ would permit the exploration of different *T*_R_ values within experimentally limited temperature ranges. For example, increasing the fatty acid tail length in the saturated, liquid-order preferring lipid of a ternary mixture typically increases $T_{mis}^{\prime}$ and *J,* while decreasing the additive’s *g* value and enhancing the additive’s phase boundary preference. Similarly, the *p* and *g* values for a membrane additive in a complex cellular membrane could be predicted by measuring the additive's effects on synthetic model membranes of greatly different molecular composition, but similar $T_{mis}^{\prime}$ and *J.*

Values of *g* and *p* that are greater than one represent the phase preferences for the additives that are stronger that then constituents of the phases themselves. For example, if dipalmitoylphosphatidylcholine (DPPC) was added to a dioleoylphosphatidylcholine (DOPC)/dimyristoylphosphatidylcholine (DMPC)/cholesterol bilayer, it would likely have a *g* > 1 since DPPC has a stronger preference for the liquid-ordered phase than DOPC, DMPC, or cholesterol. Similarly, it is feasible that a particular phase-preferring component of a hybrid molecule could have a stronger phase preference than average constituents of that phase, i.e., *p* > 1. For example, the palmitoyl group of H-Ras or palmitoyloleoylphosphatidylcholine (POPC) could prefer the liquid-ordered phase in a DOPC/DMPC/cholesterol bilayer even more than DOPC, DMPC, or cholesterol.

The quantification of *p* and *g* values of cholesterol, for example, requires knowledge of the effects cholesterol addition has on lipid phase separation and the likelihood of cholesterol to be found on the phase boundary. Cholesterol preferentially partitions into liquid-ordered phases, thereby indicating a value of *g* > 0. The addition of cholesterol to a membrane typically encourages phase miscibility, indicating that cholesterol has a value of *g* < 1. The value of *g* for cholesterol will vary with the surrounding membrane lipids; however, quantification of cholesterol’s effects on the *T*_mis_ for near-critical membranes composed of DOPC, DPPC, and cholesterol displayed approximately 15 °C reduction in *T*_mis_ with the addition of 5 mol% cholesterol (Veatch et al. 2007b), which implies a *g* value of 0.3 for cholesterol in this system (i.e., Fig. 3b). The focused quantification of changes in *T*_mis_ with variations in cholesterol concentration in near-critical ternary systems would provide greater certainty in the determination of *g* and *p*.

Similar analysis on the addition of the fluorescent lipid DiI-C12 into membranes composed of DOPC, DPPC, and cholesterol at molar ratios of 35:35:30 demonstrates how a more complex mechanism may also be present (Veatch et al. 2007a). In this system, *T*_mis_ increased linearly with increasing DiI-C12 concentrations up to 0.1 mol% with reduced effects on *T*_mis_ at higher DiI-C12 concentrations. This difference may be due to a particularly drastic effect of DiI-C12 pushing the membrane toward or away from a critical composition (Veatch et al. 2007a) or specific interactions between DiI-C12 and the other lipids in the system.

The theoretical framework presented here provides a basis for the interpretation of experimental data for the lipid phase and boundary preference of membrane-associated molecules. Similarly, this framework provides a means of predicting the lipid-raft association and lipid phase-dependent clustering in complex systems through the extension of results from model membrane studies. This ability to quantify the phase preference of membrane additives may prove to be a valuable parameter for the therapeutic development and evaluation of the therapeutic mechanisms.

## Electronic supplementary material

Below is the link to the electronic supplementary material.

Supplementary material 1 (AVI 12307 kb)

Supplementary material 2 (DOCX 19959 kb)
