# Supplementary material for: Trace membrane additives affect lipid phases with distinct mechanisms: a modified Ising model
Source: Eur Biophys J. 2015 Mar 28;44(4):227–33. doi: 10.1007/s00249-015-1017-x (PMC4412547; doi:10.1007/s00249-015-1017-x)
Supplement: Supplementary file 2 — Supplementary material 2 (DOCX 19959 kb) [file 249_2015_1017_MOESM2_ESM.docx]

**SUPPLEMENTAL MATERIAL**

**Trace membrane additives affect lipid phases with distinct mechanisms: A modified Ising model**

Rebecca L. Meerschaert and Christopher V. Kelly*

Department of Physics and Astronomy, Wayne State University, Detroit, Michigan, USA

*Address reprint requests and inquiries to Christopher V. Kelly,

Tel: 313-577-8471, Email: cvkelly@wayne.edu

**Introduction**

This Supplemental Material provides additional information about the simulation methods and results acquired from these simulations. These simulations demonstrated how trace additives of rotationally symmetric gray particles or rotationally asymmetric phase-polarized particles alter the phase mixing in a two-component Ising model. The first section, Simulation Methods, provides details on how the simulations were performed and the conditions for the simulated system. The Calculation of Miscibility Temperatures section discusses how the correlation lengths and transition temperature (*T_mis_*) were determined. This section also discusses how the resulting correlation length plotted against the reduced temperature (*T_R_*) of the system can determine *T_mis_*. The System Configurations section includes analysis of the system morphology versus temperature, additive concentraiton, and additive polarization or gray values. Insets of the images are included to improve the understanding of the individual additive locations within a phase or at the boundary between phases.

**Simulation Methods**

Computational simulations were performed in custom written C code that was compiled with MinGW and executed in batch through MATLAB (The MathWorks, Inc.) with inspiration taken from previous uses of Ising models to study membranes (Jorgensen et al. 1991; Frazier et al. 2007; Honerkamp-Smith et al. 2008; Machta et al. 2011). The C code loaded a starting configuration of 512 × 512 particles in a square lattice, which was close to a minimized internal energy configuration with the desired fraction of included additives. The C code then iteratively performed particle exchange sweeps during which each particle in the system was potentially exchanged with another randomly chosen particle in the system with conserved order parameter with biperiodic boundary conditions (i.e., 512 × 512 = 262,144 potential particle exchanges per sweep). Although these non-local particle exchanges do not reconstruct system dynamics accurately, they do equilibrate to representative system configurations with much improved speed over methods that only permit nearest-neighbor exchanges (Machta et al. 2011).

Whether or not a particular particle exchange was performed depended on the change in system energy upon particle exchange (*ΔH, Eq. 1*). If *ΔH* ≤ 0 for the exchange of two randomly chosen particles, then the particle positions were exchanged. If *ΔH* > 0, then the particles were exchanged only if a random number chosen between zero and one happened to be larger than exp(-*ΔH*/(*k_B_T*)), as is convention for Monte Carlo algorithms. After every 2000 sweeps, the system configuration was saved and analyzed. Custom-made MATLAB code iteratively executed this C code and facilitated looping over varying temperature, additive fraction, and additive properties (i.e., *p* and *g*). Each configuration was run for 2 × 10^5^ sweeps with the first 10^5^ sweeps used solely for system equilibration.

When rotationally asymmetric particles were included in the simulation (i.e., *p* ≠ 0), then an additional step was added into the computational routine to permit particle rotation with similar considerations of the energy differences upon particle rotation. For every sweep, the configuration's internal energy was compared with and without the rotation of each rotationally asymmetric particle of either 90°, 180°, or 270°. As with particle position exchanges, if a particle rotation resulted in a decrease of the internal energy or a sufficiently large random number was chosen, then the particle was rotated.

With the exception of systems with additives of *p* or *g* = 4, the internal energy, phase separation, and correlation lengths were equilibrated after 10^4^ loops. When *p* or *g* = 4, the white and black particles became well equilibrated, but the structure of the additive aggregates did not. The images presented in this manuscript are the result of the concluding configuration for each simulation condition after 2 × 10^5^ sweeps. Correlation lengths presented in this manuscript are the average for each simulation condition over 10^5^ sweeps after 10^5^ sweeps of equilibration.

**Calculation of Miscibility Temperatures**

The phase correlation length (*ξ*) was calculated, as explained in the manuscript (Eq. 2-5). A translation of *ξ* versus *T_R_* to maximize overlap quantifies *ΔT_mis_* between the differing systems (Figs. 2-5, S1, S2). Further, the transition temperature of each system was calculated based on the temperature at which the specific heat was a maximum (Fig. S3). This calculation of *T_mis_* for each system provided agreement with the assessment of *T_mis_* from correlation lengths within uncertainty with the translation of correlations lengths proved to be more robust method of determining *ΔT_mis_*.

**System Configurations**

The system morphology with varying temperature, additive fraction (Fig. S5), gray value (Fig. S6), and polarization (Fig. S6) demonstrate the partitioning of the additives within the system and the phase miscibility. Additionally, the supplemental movie demonstrates 50 different time points analyzed for Fig. 2. Phase mixing was enhanced with increasing temperature but could increase or decrease with additive addition depending on the additive properties. Additionally, these system configurations show how some additives are more likely to be located at the phase boundary, while others are more likely to be segregated into one particular phase. When *p* or *g* were greater than 3, the additives aggregated and had a reduced effect on the white-black phase mixing (Fig. S6, S7). The additive aggregates for phase polarized particles with *p* = 4 demonstrate locally ordered orientations to ensure all additive edges were interacting with their preferred neighboring surface (Fig. S7). This requirement of proper particle rotation slowed the diffusion of the phase polarized additives within their aggregates. The aggregates composed solely of additives with large *g* values were a fluid phase, as would be expected for this Monte Carlo simulation that would allow two gray particles to exchange locations as likely as two white particles to exchange locations since there would be no energy difference in the configuration up on exchange of any of these rotationally symmetric particles.


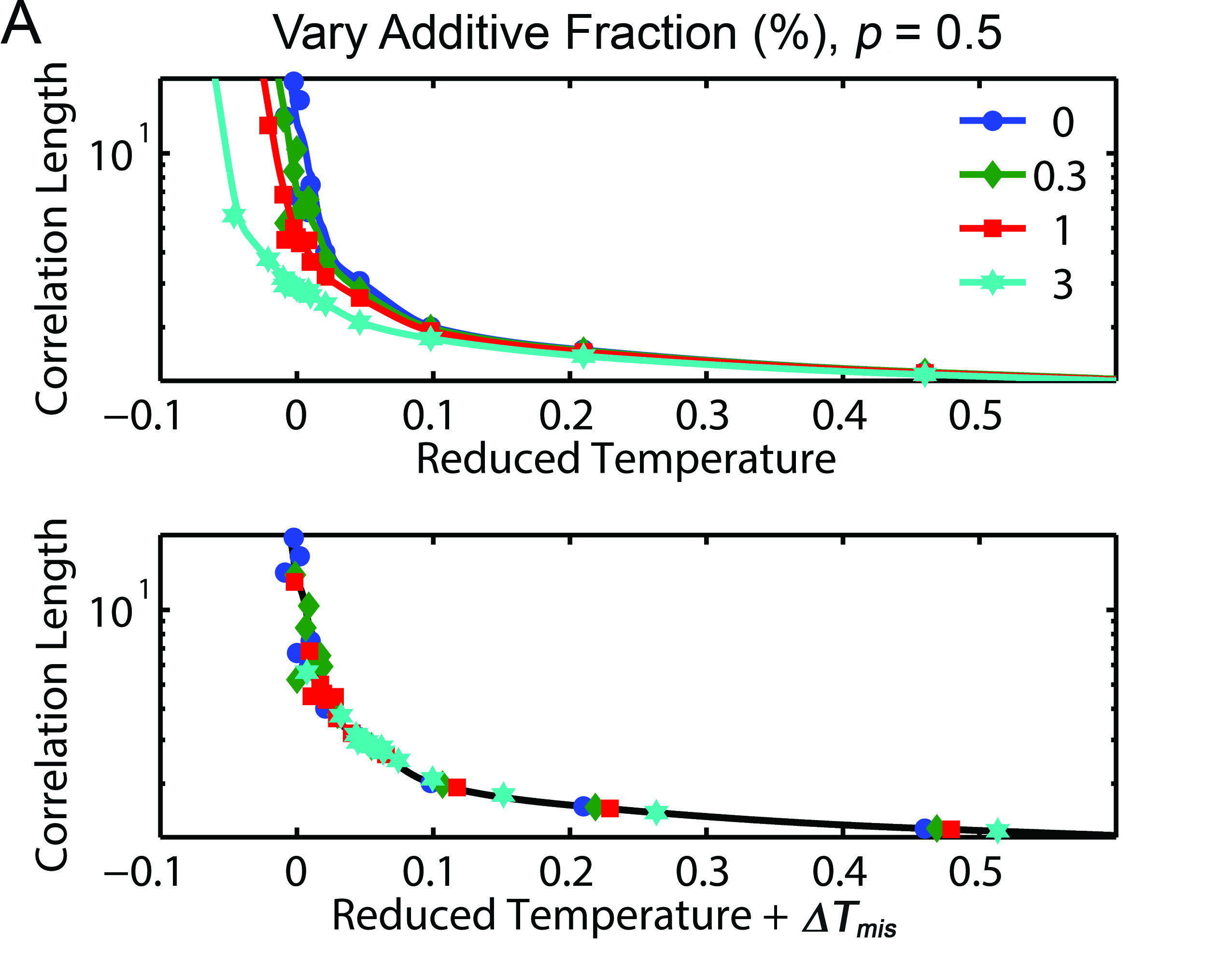

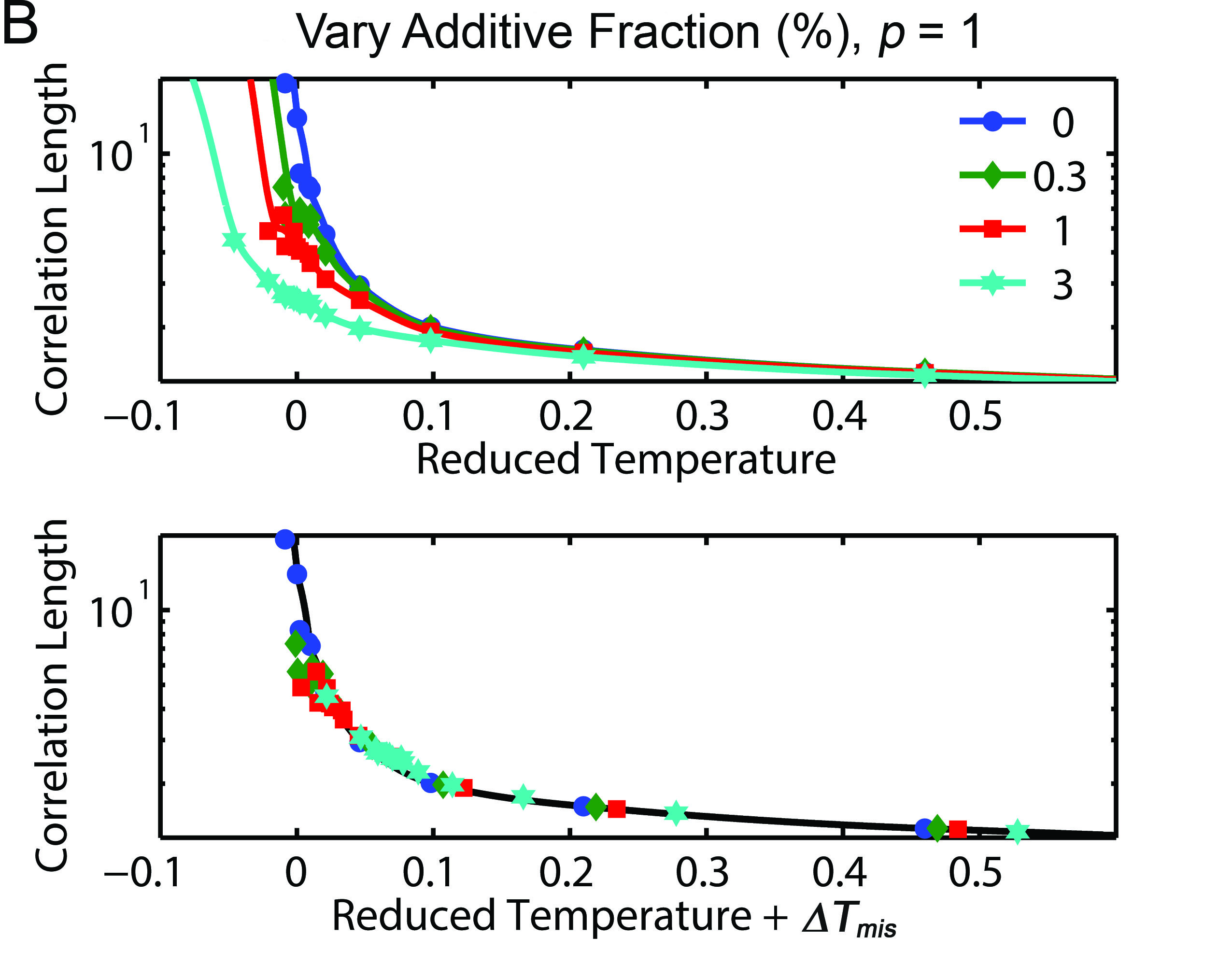


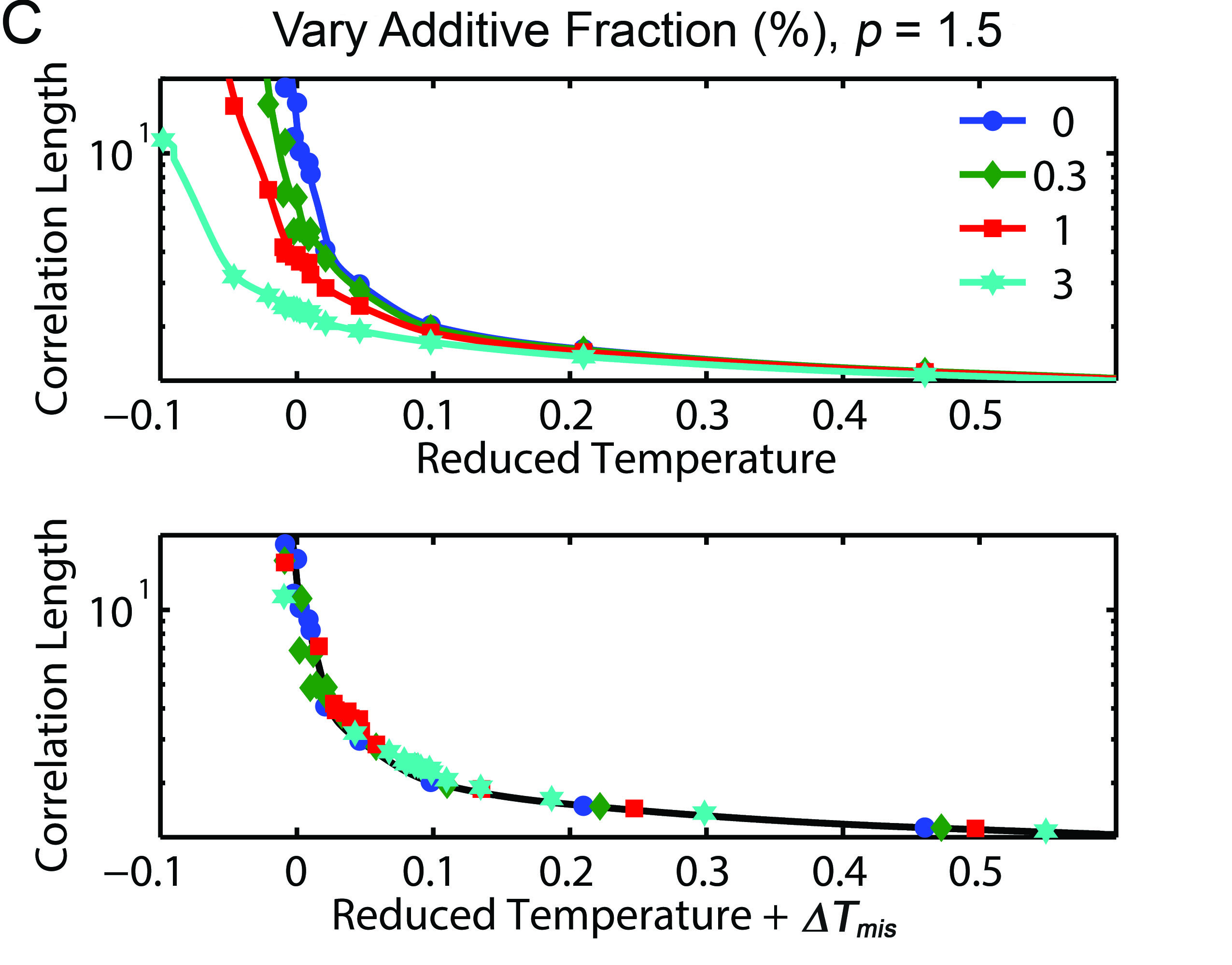


**Figure S1** A constant temperature offset was determined for each simulated system to achieve overlap of the correlation length versus temperature, also known as the change in miscibility temperature (*ΔT_mis_*). The phase correlation length versus temperature was consistent for each simulations system with the appropriate temperature scaling by *ΔT_mis_*. The initial and scaled correlation length versus reduced temperature results for (A) *p* = 0.5, (B) *p* = 1, and (C) *p* = 1.5 are shown here with varying additive fractions: 0, 0.3, 1 and 3 mol%.


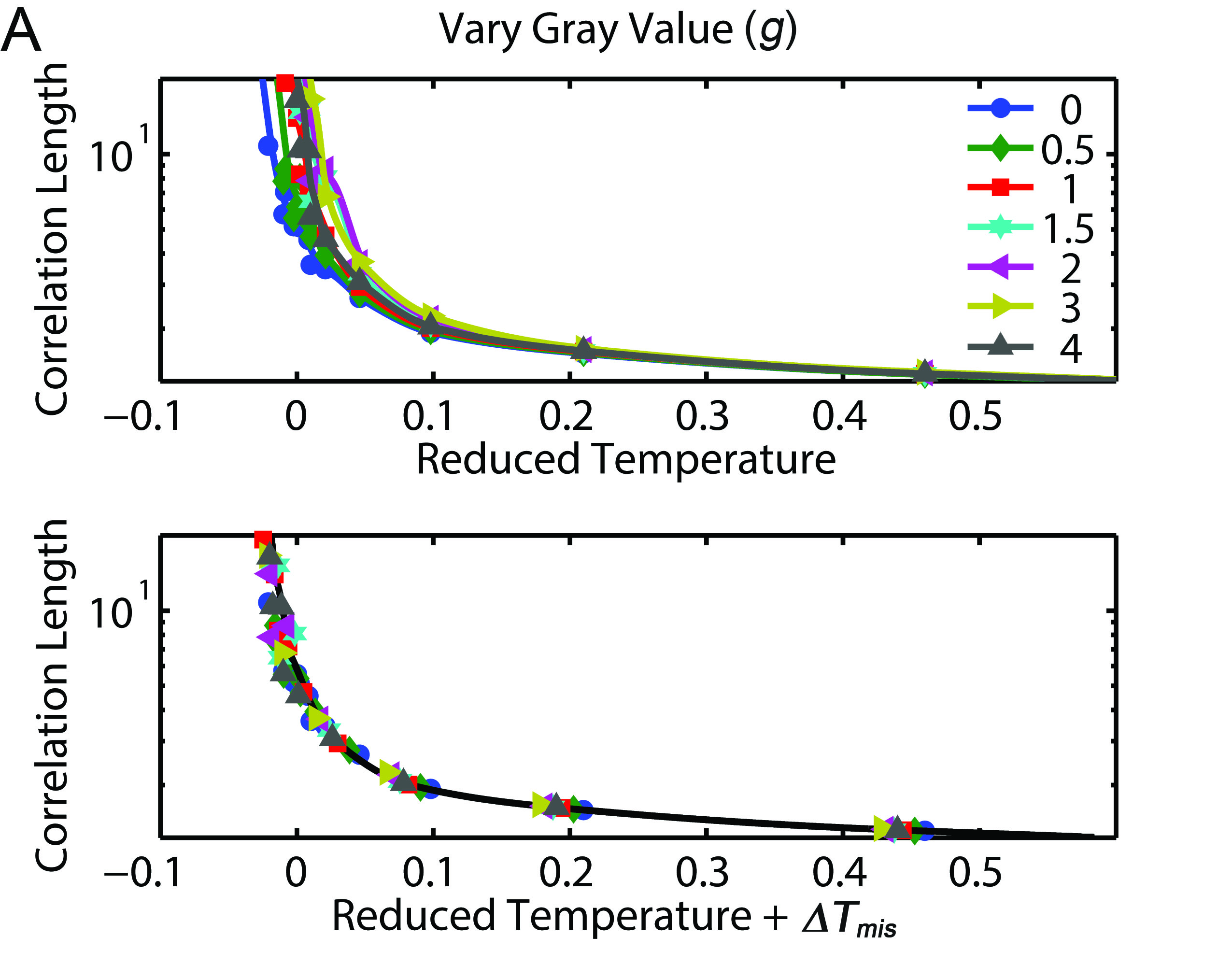

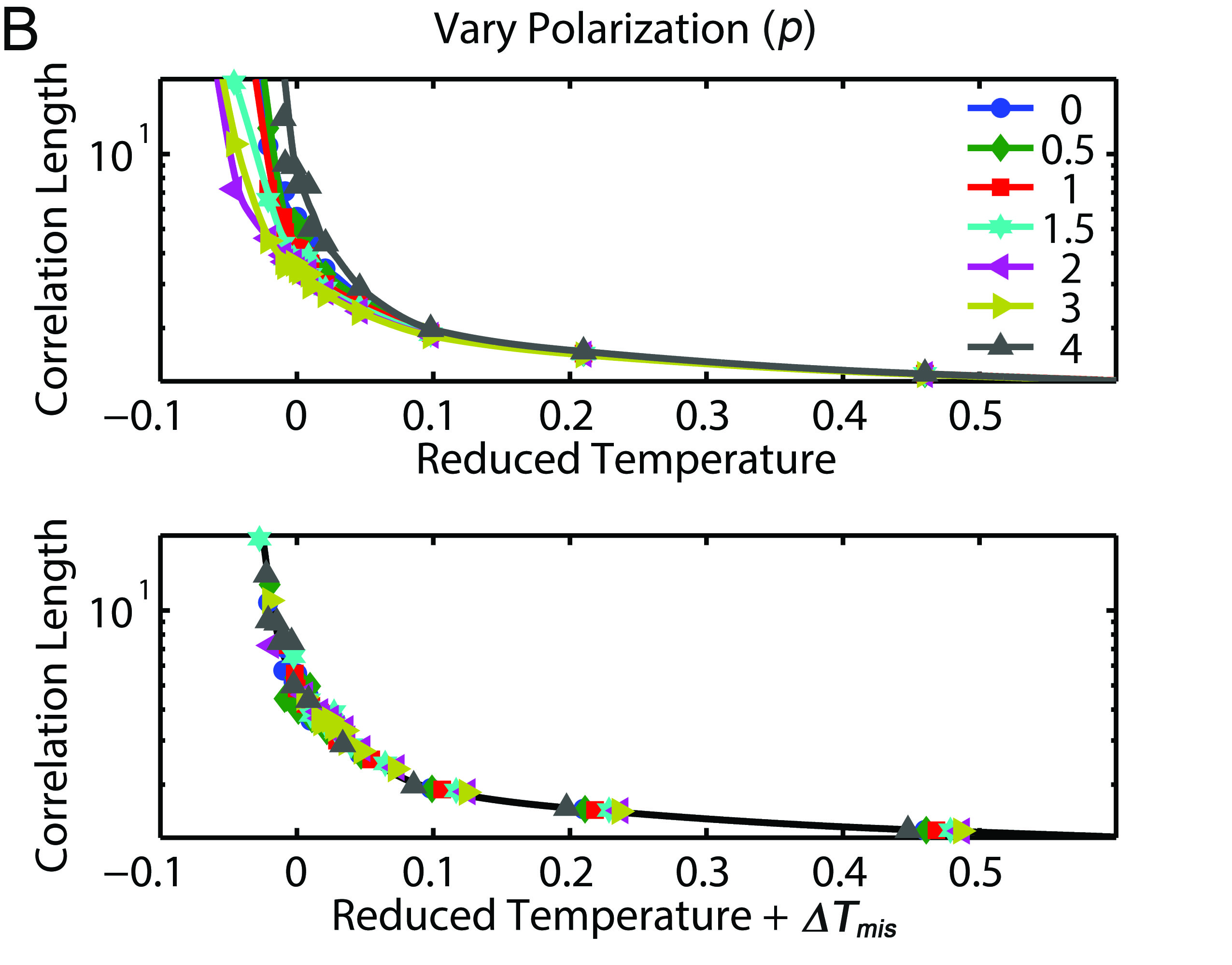


**Figure S2** Determining a constant temperature to add to each plot of *ξ* versus *T_R_* to maximize overlap permitted quantification of *ΔT_mis_* for varying gray value and polarization. The top row shows the raw simulation result of correlation length (ξ) versus reduced temperature. The bottom row shows the translated temperature of additive type to maximize overlap and determine *ΔT_mis_*. All of these systems contained 1 mol% additives.


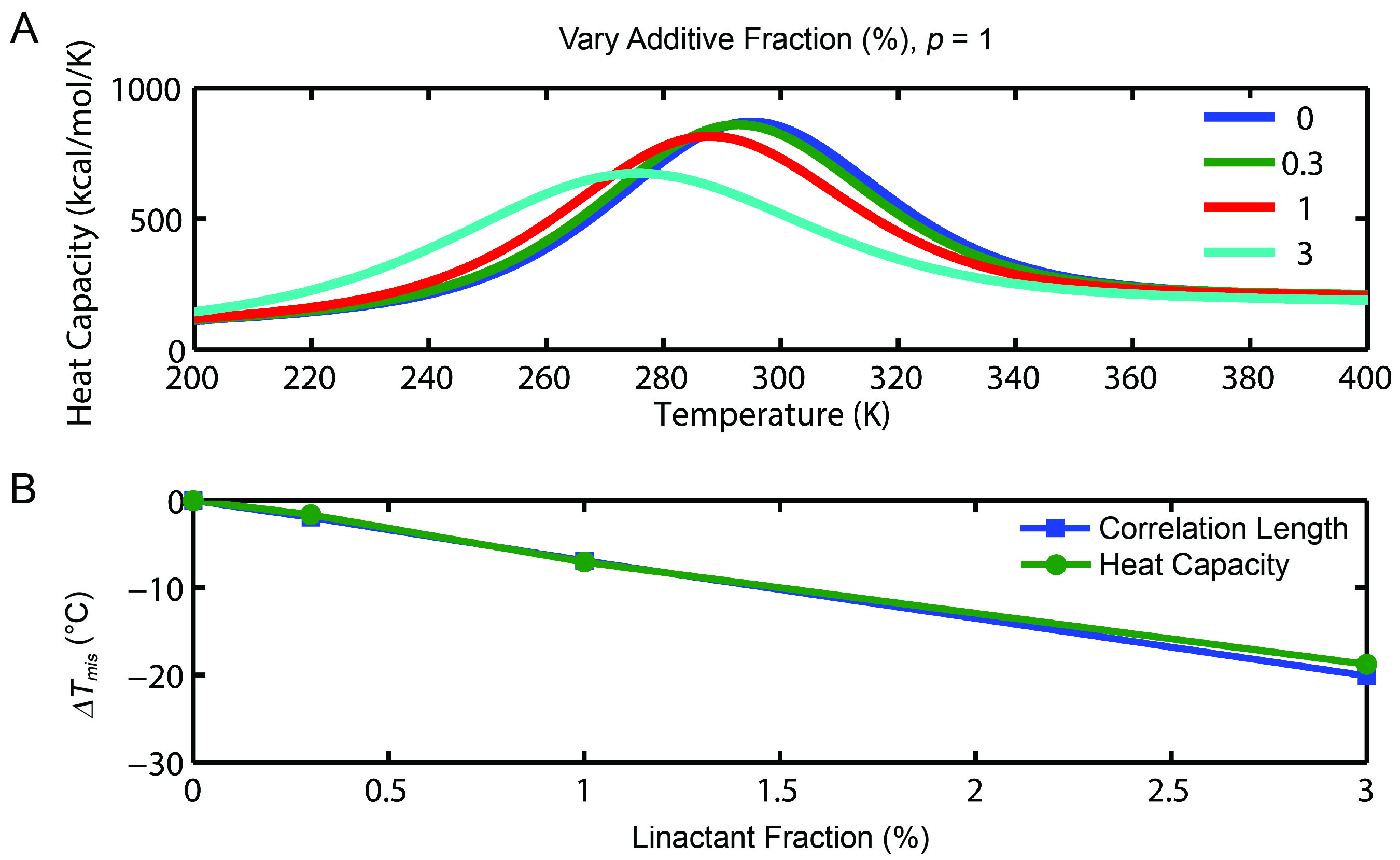


**FIGURE S3** (A) The changes in transition temperature were also assessed by observing the specific heat at varying temperatures. Shown here are fits to the heat capacity versus temperature for determination of the temperature maximum of the heat capacity. (B) The phase transition temperature determined at the maximum of the specific heat showed high correlation to the phase transition temperature determined by the temperature shift necessary to achieve matching correlation lengths versus temperature. Shown here are the results for additives of *p* = 1 with varying additive fractions: 0, 0.3, 1 and 3 mol%. Determining *ΔT_mis_* _­_from shifting *ξ* proved to be more consistent than peak identification from heat capacity changes.


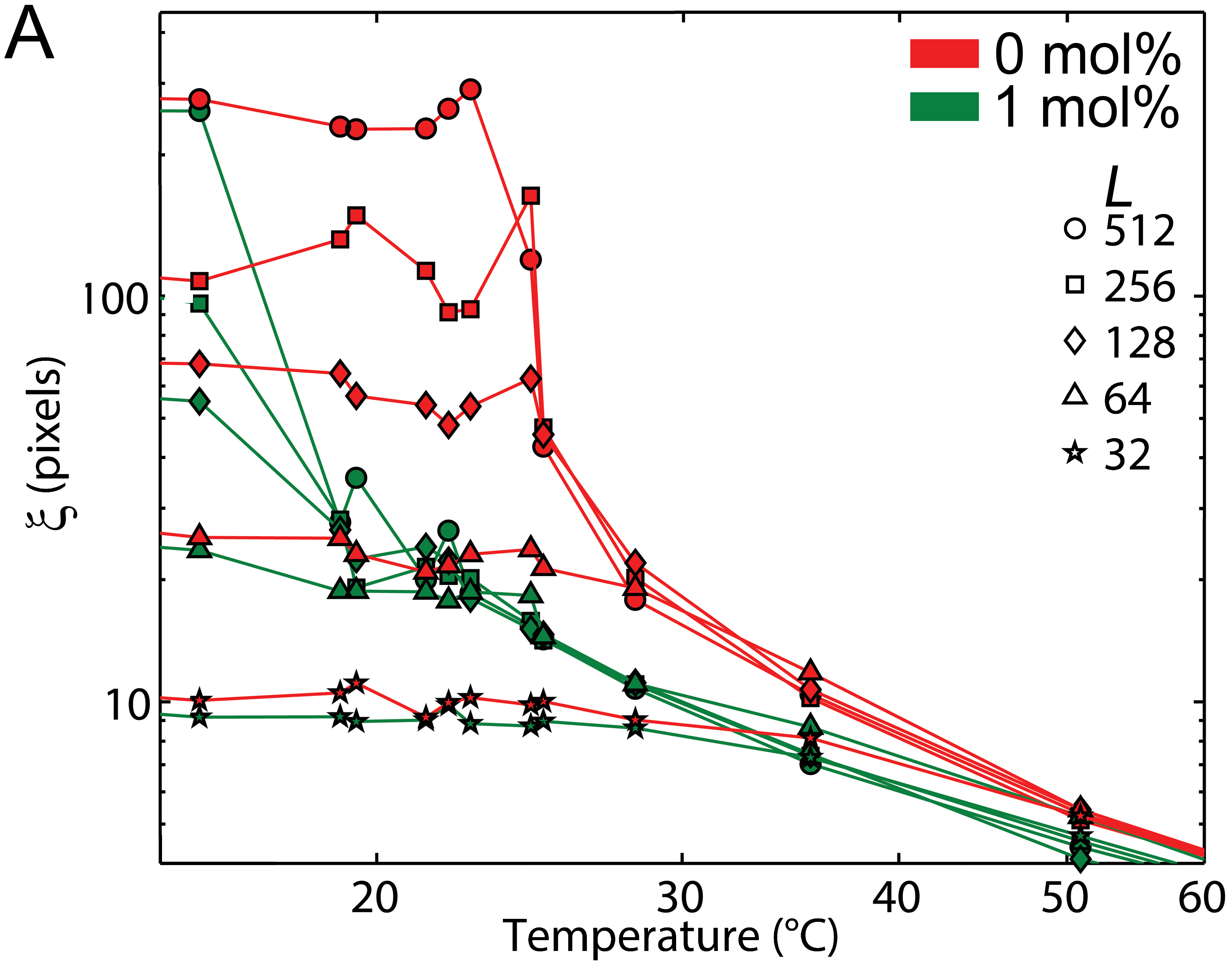

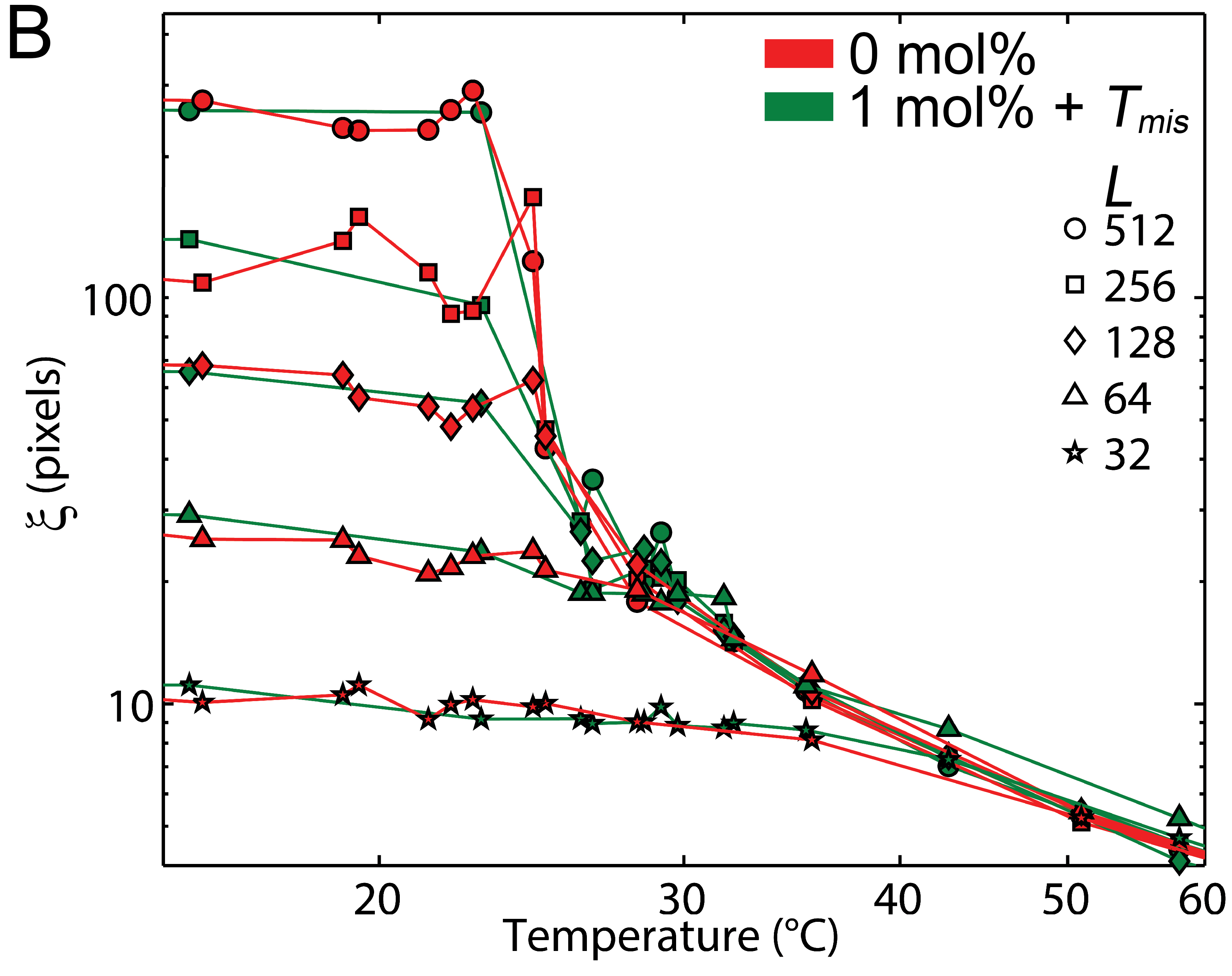


**FIGURE S4** The system size (*L*) limits the maximum *ξ* that can be observed. Simulation results presented throughout this manuscript were calculated from a 512x512 square lattice, i.e., *L =* 512, with the exception of the results presented in this figure. Increasing *L* permits greater range of observed *ξ*; however, the effects of changing additive concentration are consistent within systems of the same *L*. This is demonstrated in *ξ* versus temperature (A) without and (B) with translating the data for 1 mol% phase-polarized particle addition (*p* = 1) data along the temperature axis by *T_mis_* = 7.4 °C.

**
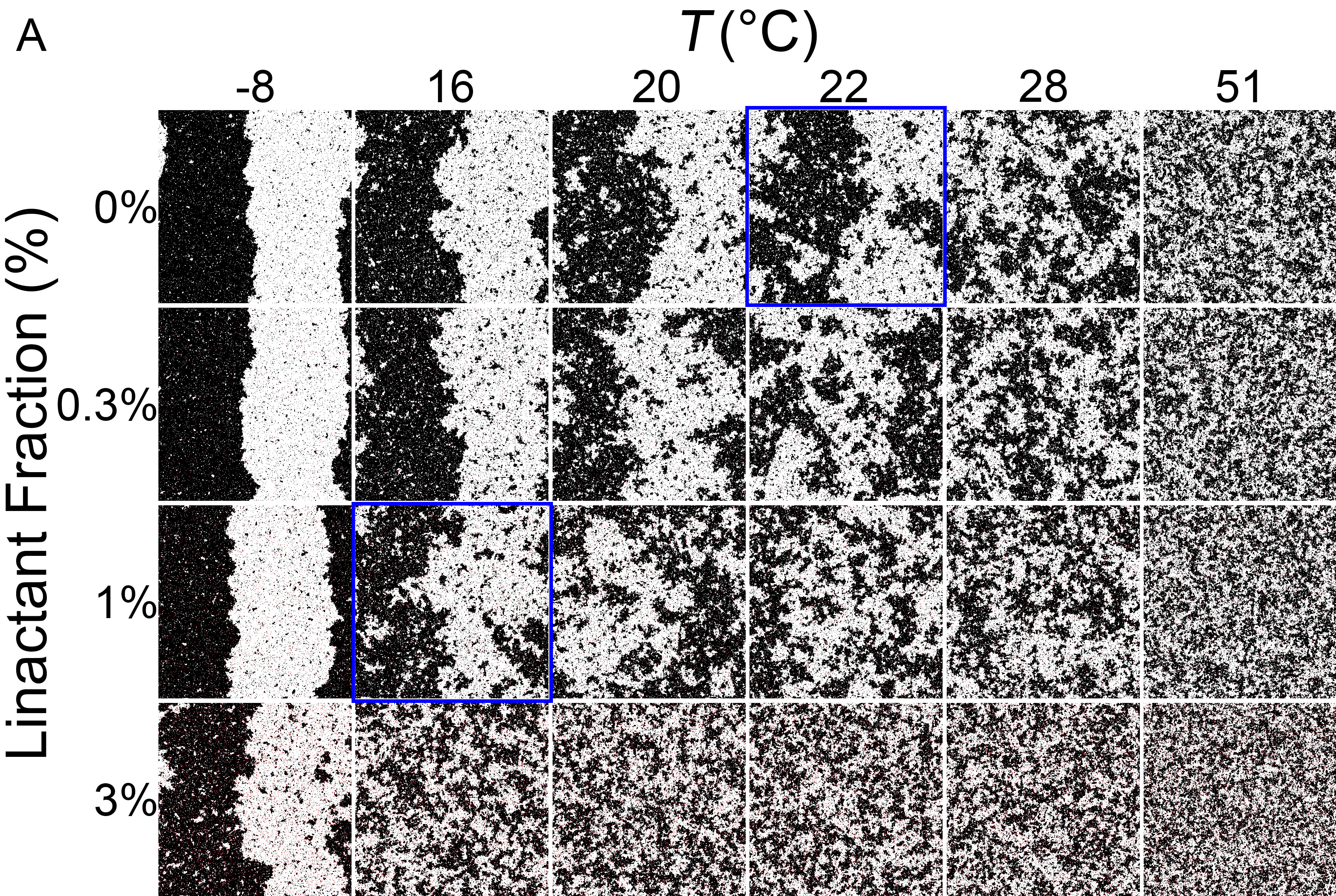

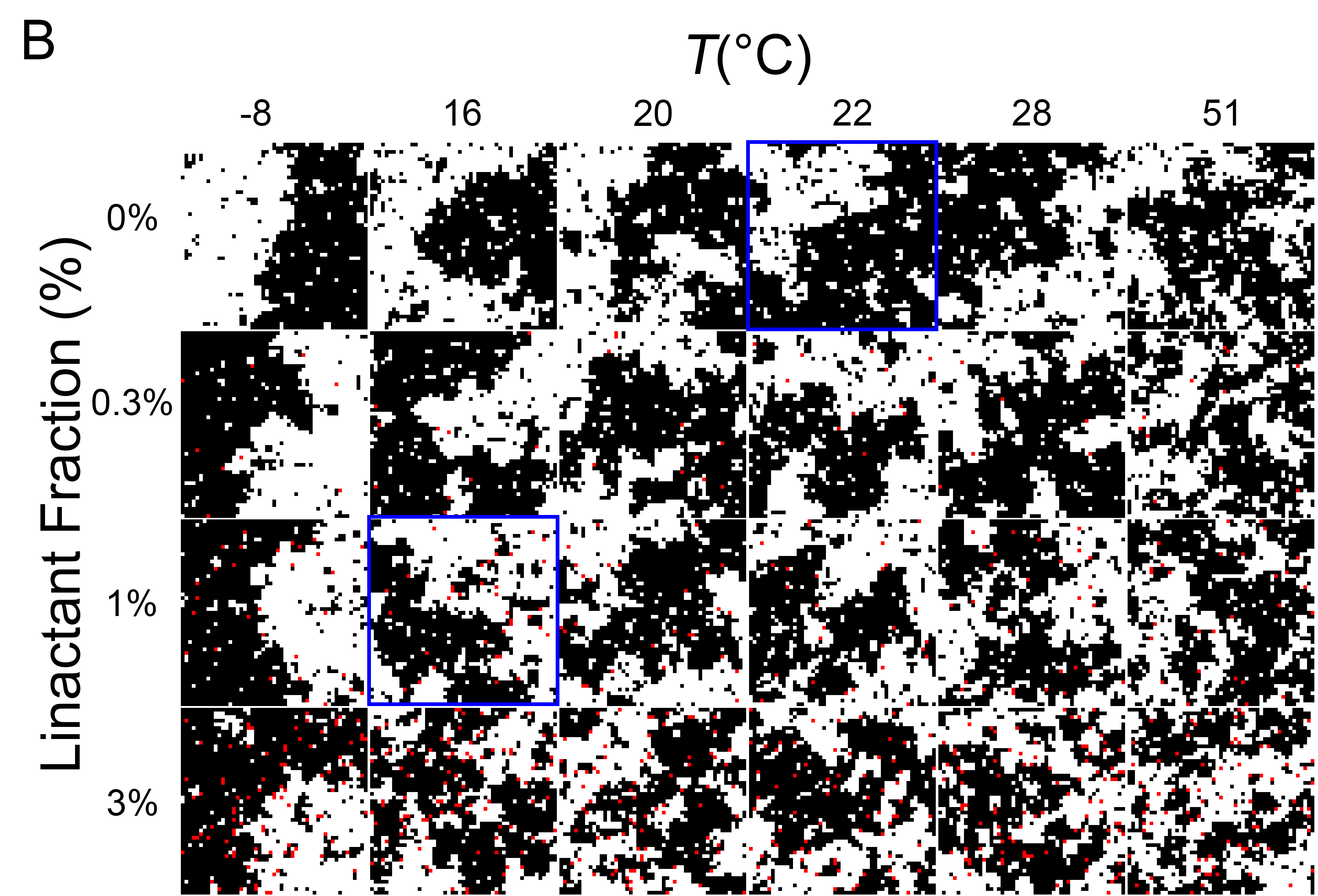
**

**FIGURE S5** Increasing the fraction of phase-polarized particles with a polarization of *p* = 1 resulted in a decrease of the correlation length and a decrease in the miscibility transition temperature. The blue outlined images represent the miscibility temperature for each change in fraction of the boundary-active particles. In (A) the full 512 x 512 configuration and (B) the zoomed-in 50 x 50 regions, the phase-polarized particles are displayed as red squares.


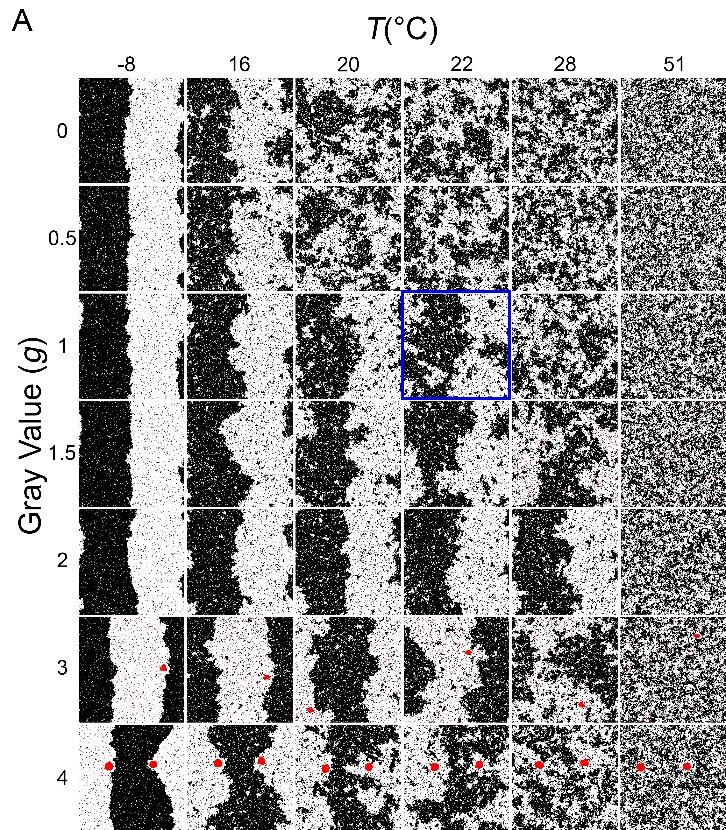

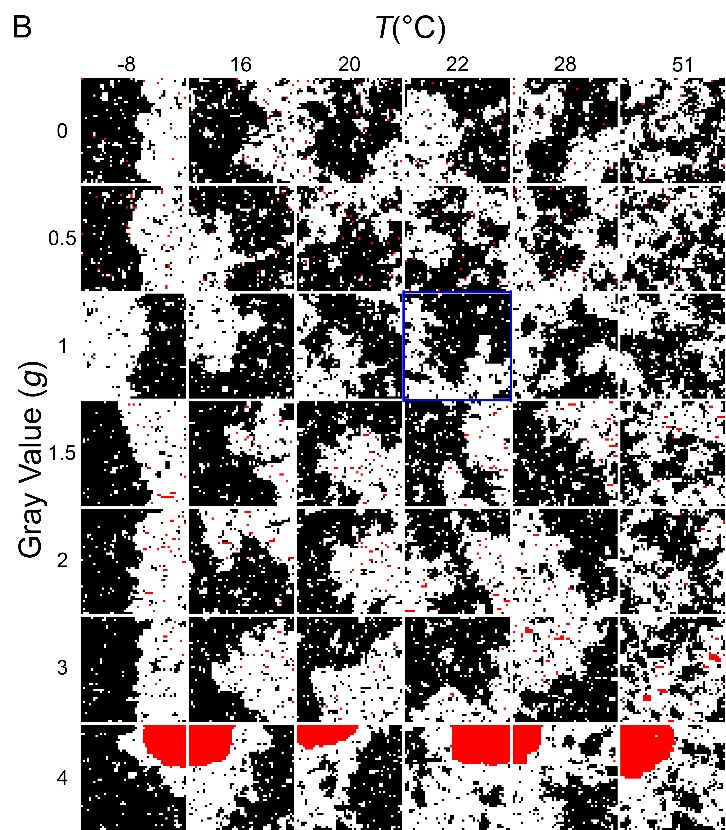

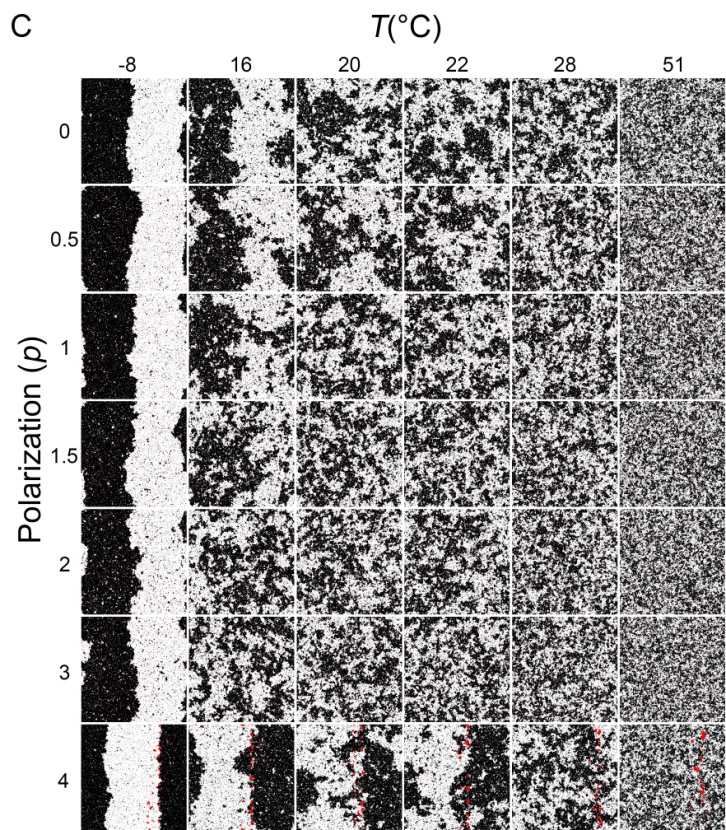

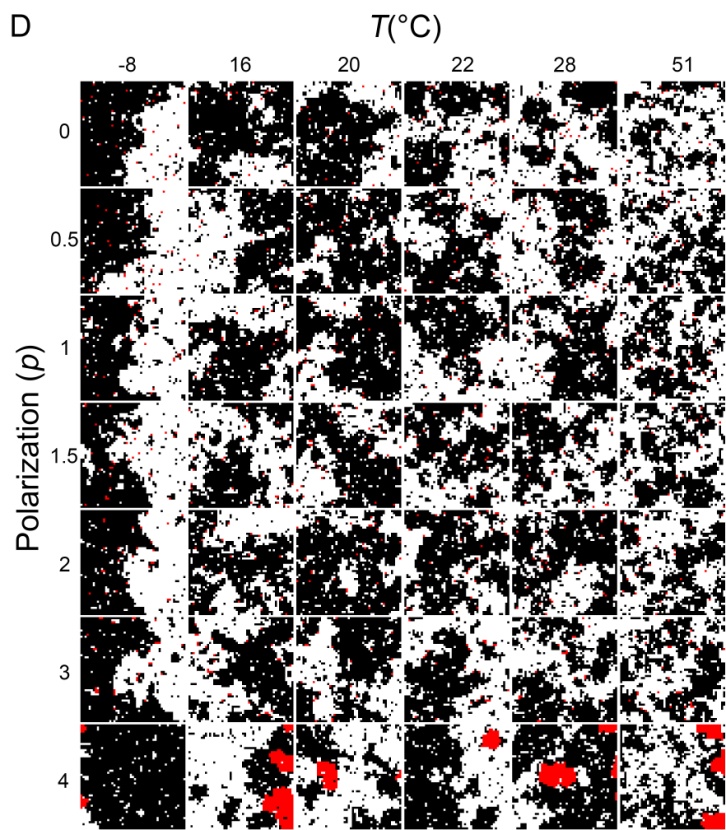


**FIGURE S6** Increasing the (A, B) gray value or (C, D) polarization of the additives altered the phase mixing and additive aggregation with 1 mol% additive. In (A, C) the final 512 x 512 configuration and (B, D) zoomed-in 50 x 50 regions, the phase-polarized particles are displayed as red squares. When *p* or *g* > 3, the additives condensed and formed a distinct phase excluding white or black particles (Fig. S7). The blue outlined images represent the configurations at their miscibility temperature. When *g* = 1 the additives were equivalent to white particles and the system did not display a difference in miscibility temperature from the additive-free configuration (Fig. 3). When *p* or *g* > 3, simulations were unable to capture a diverse set of configurations for the additive aggregation due to the improbable rearrangement of the condensed phases.


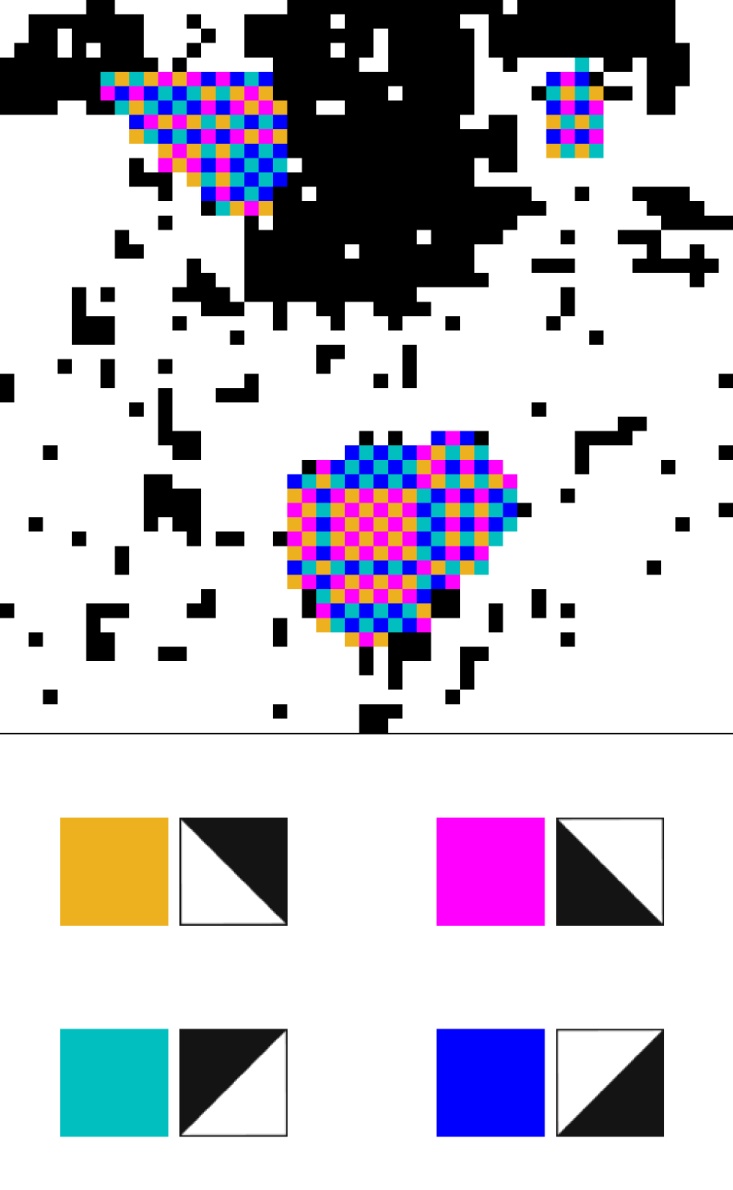


**FIGURE S7** When *p* ≥ 4, the additives aggregated into a distinct ordered phase excluding the white and black particles. Shown here is an inset from the system configuration (*top*) equilibrated for 2 × 10^5^ sweeps with 1 mol% phase-polarized particles of *p* = 4 at 22°C where the phase-polarized particles are color-coded (*bottom*) to demonstrate their orientation. All phase-polarized particle edges are interfacing with their preferred phase. Longer equilibrations would have likely resulted in the aggregations clustering to grow in size and reduce in number.

**
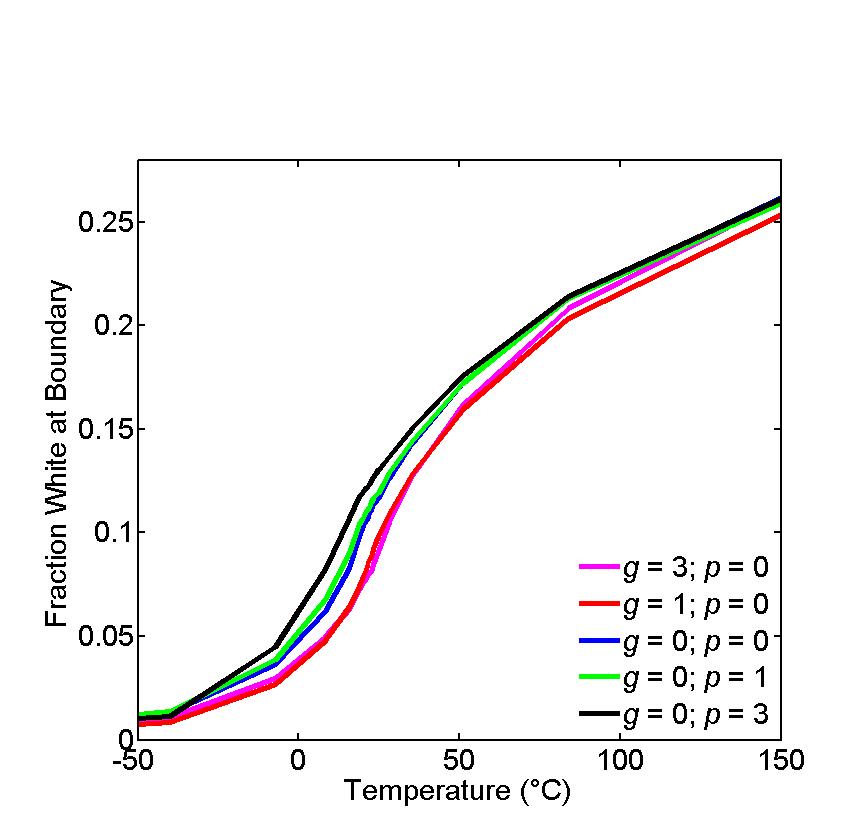
**

**FIGURE S8** Higher temperatures result in greater phase mixing and more phase boundary distance within the system. Here the number of white particles that were at the phase boundary (i.e., adjacent to two black particles and two adjacent particles) are compared to the number of all white particles, excluding the white particles that are adjacent to an additive.

**SUPPORTING REFERENCES**

Frazier ML, Wright JR, Pokorny A, Almeida PFF (2007) Investigation of domain formation in sphingomyelin/cholesterol/POPC mixtures by fluorescence resonance energy transfer and Monte Carlo simulations. Biophys J 92:2422–2433. doi: 10.1529/biophysj.106.100107

Honerkamp-Smith AR, Cicuta P, Collins MD, et al (2008) Line tensions, correlation lengths, and critical exponents in lipid membranes near critical points. Biophys J 95:236–246. doi: 10.1529/biophysj.107.128421

Jorgensen K, Ipsen J, Mouritsen O, et al (1991) The Effects of Density-Fluctuations on the Partitioning of Foreign Molecules into Lipid Bilayers - Application to Anesthetics and Insecticides. Biochim Biophys Acta 1067:241–253. doi: 10.1016/0005-2736(91)90050-I

Machta BB, Papanikolaou S, Sethna JP, Veatch SL (2011) Minimal model of plasma membrane heterogeneity requires coupling cortical actin to criticality. Biophys J 100:1668–1677. doi: 10.1016/j.bpj.2011.02.029
